# Effect of Myopic Defocus on Visual Acuity after Phakic Intraocular Lens Implantation and Wavefront-guided Laser *in Situ* Keratomileusis

**DOI:** 10.1038/srep10456

**Published:** 2015-05-21

**Authors:** Kazutaka Kamiya, Kimiya Shimizu, Akihito Igarashi, Takushi Kawamorita

**Affiliations:** 1 Department of Ophthalmology, Kitasato University School of Medicine, Kanagawa, Japan; 2 Kitasato University School of Allied Health Sciences, Kanagawa, Japan

## Abstract

This study aimed to investigate the effect of myopic defocus on visual acuity after phakic intraocular lens (IOL) implantation and wavefront-guided laser *in situ* keratomileusis (wfg-LASIK). Our prospective study comprised thirty eyes undergoing posterior chamber phakic IOL implantation and 30 eyes undergoing wfg-LASIK. We randomly measured visual acuity under myopic defocus after cycloplegic and non-cycloplegic correction. We also calculated the modulation transfer function by optical simulation and estimated visual acuity from Campbell & Green’s retinal threshold curve. Visual acuity in the phakic IOL group was significantly better than that in the wfg-LASIK group at myopic defocus levels of 0, –1, and –2 D (p < 0.001, p < 0.001, and p = 0.02, Mann-Whitney U-test), but not at a defocus of –3 D (p = 0.30). Similar results were also obtained in a cycloplegic condition. Decimal visual acuity values at a myopic defocus of 0, −1, −2, and -3 D by optical simulation were estimated to be 1.95, 1.21, 0.97, and 0.75 in the phakic IOL group, and 1.39, 1.11, 0.94, and 0.71 in the wfg-LASIK group, respectively. From clinical and optical viewpoints, phakic IOL implantation was superior to wfg-LASIK in terms of the postoperative visual performance, even in the presence of low to moderate myopic regression.

Laser *in situ* keratomileusis (LASIK) is now extensively recognized as a predictable and effective refractive surgical procedure for the correction of myopia and myopic astigmatism. Although corneal ectasia is known to be a multifactorial disease, patients with high myopia or thin corneas face some restrictions in avoiding the possible risk of developing ectasia. Since LASIK requires more laser ablation in highly myopic eyes, the cornea becomes more oblate, resulting in more surgically induced higher-order aberrations (HOAs), especially spherical aberrations, which may lead to deteriorate intrinsic corneal optical performance^1^,^2^ The Visian Implantable Collamer Lens (ICL^TM^, STAAR Surgical, Nidau, Switzerland), a posterior chamber phakic intraocular lens (IOL), reportedly offers effective correction of moderate to high ametropia^3^,^4^,^5^,^6^,^7^,^8^,^9^,^10^,^11^,^12^,^13^,^14^ ICL implantation has been demonstrated to be superior in the great majority of the measures of safety, efficacy, predictability, and stability to conventional or wavefront-guided LASIK (wfg-LASIK) in eyes with all degrees of myopia, from low to high^15^,^16^,^17^,^18^

If myopic regression of the initial surgical effect takes place, the predictability, efficiency, and stability of refractive surgery may all be affected, so that visual performance deteriorates and the patient becomes dissatisfied. Some long-term regression has been observed, not only after LASIK, but also after ICL implantation^19^,^20^,^21^,^22^,^23^,^24^,^25^ However, to our knowledge, the circumstances regarding myopic defocus after either surgical procedure have not so far been quantitatively investigated. Myopic defocus from the targeted refraction plays an essential role, not only in refractive and visual outcomes themselves, but also in patient satisfaction, following these surgical procedures. The present study is intended, from both clinical and optical viewpoints, to prospectively assess the effects of myopic defocus on visual outcomes after ICL implantation and wfg-LASIK for the correction of myopia and myopic astigmatism.

## Results

### Patient Population

Preoperative demographics of the study population are summarized in Table 1. All surgeries were uneventful, and no vision-threatening complications were seen throughout the observation period. There were no significant differences between the two groups in terms of age (p = 0.27, Mann-Whitney U test), gender (p = 0.38), preoperative logMAR (logarithm of the minimum angle of resolution) corrected distance visual acuity (CDVA) (p = 0.53), preoperative cylinder (p = 0.41), postoperative logMAR uncorrected distance visual acuity (UDVA) (p = 0.11), postoperative spherical equivalent (p = 0.98), or postoperative cylinder (p = 0.94), although there were significant differences in terms of preoperative logMAR UDVA (p < 0.001), preoperative spherical equivalent (p < 0.001), and postoperative logMAR CDVA (p = 0.01).

### Corrected Visual Acuity under Myopic Defocus

**Figure 1:**
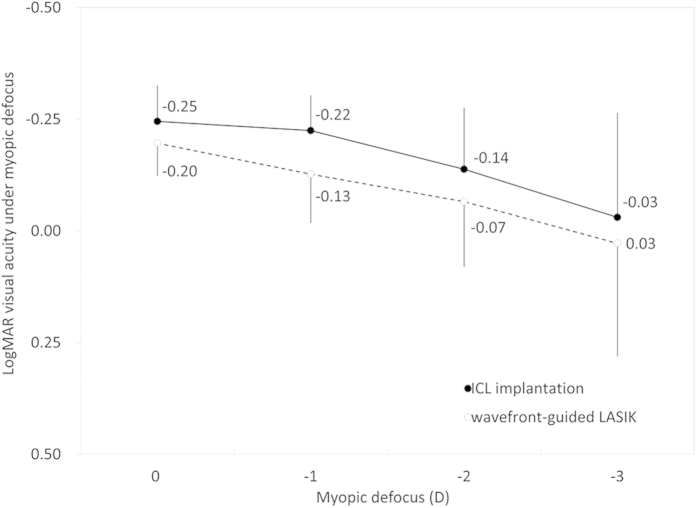
LogMAR (logarithm of the minimum angle of resolution) visual acuity under myopic defocus of 0, −1, −2, and -3 D in a non-cycloplegic condition in the implantable collamer lens (ICL) and wavefront-guided laser *in situ* keratomileusis (wfg-LASIK) groups.

LogMAR visual acuity under myopic defocus of 0, –1, –2, and –3 D was –0.25 ± 0.08, –0.22 ± 0.08, –0.14 ± 0.14, and –0.03 ± 0.23 in the ICL group, and –0.20 ± 0.07, –0.13 ± 0.11, –0.07 ± 0.15, and 0.03 ± 0.25 in wfg-LASIK group, respectively, in a non-cycloplegic condition (Fig. 1). The variance of the data was statistically significant in both groups (p < 0.001, One-way ANOVA). Multiple comparisons demonstrated significant differences between measurements made without myopic defocus and with myopic defocus of –2 and –3 D (p = 0.01 and p < 0.001, respectively), and no significant differences between measurements made without myopic defocus and with a defocus of –1 D (p = 0.90) in the ICL group. Multiple comparisons demonstrated significant differences between measurements made without myopic defocus and with myopic defocus of –2 and –3 D (p = 0.002 and p < 0.001, respectively), and no significant differences between measurements made without myopic defocus and with a defocus of –1 D (p = 0.09) in the wfg-LASIK group. We found significant differences in logMAR visual acuity at myopic defocus levels of 0, –1, and –2 D (p < 0.001, p < 0.001, and p = 0.02, respectively, Mann-Whitney U-test), but no significant difference at a defocus of –3 D (p = 0.30), between the two groups.

**Figure 2:**
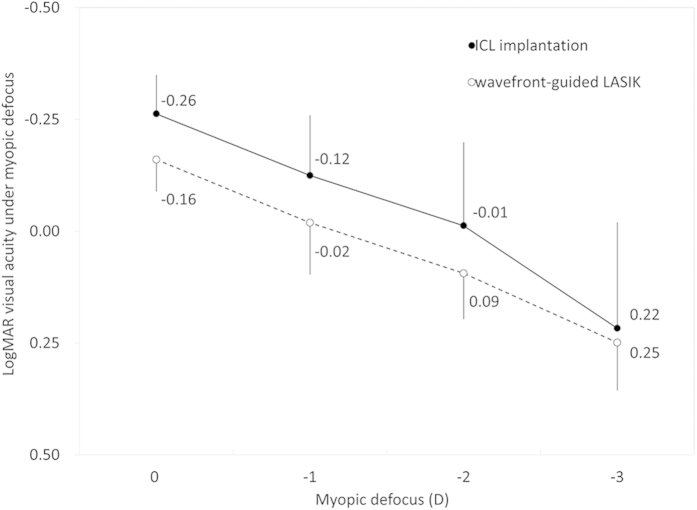
LogMAR (logarithm of the minimum angle of resolution) visual acuity nder myopic defocus of 0, –1, –2, and –3 D in a cycloplegic condition using a 3-mm artificial pupil in the implantable collamer lens (ICL) and wavefront-guided laser *in situ* keratomileusis (wfg-LASIK) groups.

Similarly, logMAR visual acuity under myopic defocus of 0, –1, –2, and –3 D was –0.26 ± 0.09, –0.12 ± 0.13, –0.01 ± 0.19, and 0.22 ± 0.24 in the ICL group, and –0.16 ± 0.07, –0.02   ± 0.11, 0.09 ± 0.10, and 0.25 ± 0.11 in wfg-LASIK group, respectively in a cycloplegic condition using a 3-mm artificial pupil (Fig. 2). The variance of the data was statistically significant in both groups (p < 0.001, One-way ANOVA). Multiple comparisons demonstrated significant differences between measurements made without myopic defocus and with myopic defocus of –2 and –3 D (p < 0.001), and no significant differences between measurements made without myopic defocus and with a defocus of –1 D (p = 0.05) in the ICL group. Multiple comparisons demonstrated significant differences between measurements made without myopic defocus and with myopic defocus of –2 and –3 D (p < 0.001), and no significant differences between measurements made without myopic defocus and with a defocus of –1 D (p = 0.14) in the wfg-LASIK group. We found significant differences in logMAR visual acuity at myopic defocus levels of 0, –1, and –2 D (p < 0.001, p = 0.008, and p = 0.002, respectively, Mann-Whitney U-test), but no significant difference at a defocus of -3 D (p = 0.42), between the two groups.

### Optical Simulation of Modulation Transfer Function and Visual Acuity under Myopic Defocus

**Figure 3:**
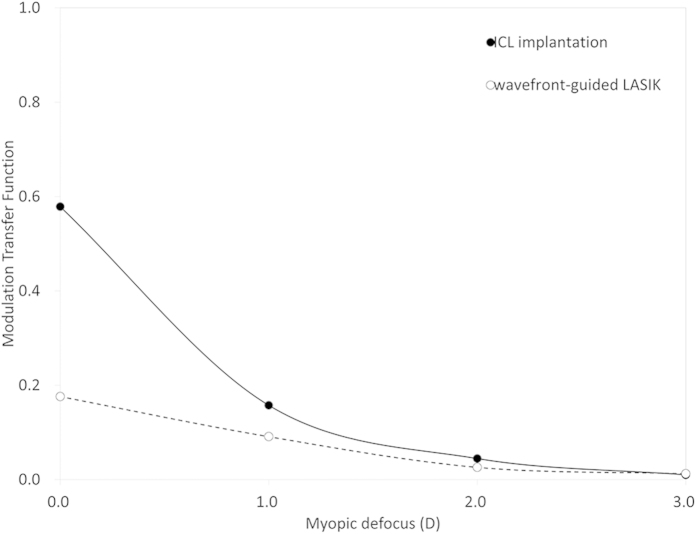
The modulation transfer function curves at a myopic defocus of 0,−1, −2, and −3 D by optical simulation at 60 cycles/mm in the implantable collamer lens (ICL) and wavefront-guided laser *in situ* keratomileusis (wfg-LASIK) groups.

The MTF curves at a myopic defocus of 0, –1, –2, and –3 D by optical simulation at 60 cycles/mm in the ICL and wfg-LASIK groups were shown in Fig. 3. For a 3.0-mm pupil, the estimated decimal visual acuity at a myopic defocus of 0, –1, –2, and –3 D by optical simulation was 1.95, 1.21, 0.97, and 0.75 in the ICL group, and 1.39, 1.11, 0.94, and 0.71 in wfg-LASIK group, respectively.

## Discussion

The results of the current study revealed that ICL implantation provided better visual outcomes than wfg-LASIK, even when low to moderate myopic regression occurred after surgery in a clinical setting. The study’s results were supported by estimates of visual acuity after ICL implantation and wfg-LASIK by optical simulation. Our results also demonstrated that visual acuity under a non-cycloplegic condition was better than that under a cycloplegic condition in each group, especially when the amount of myopic defocus is large. This discrepancy in visual acuity under cycloplegic and non-cycloplegic conditions may be largely attributed to the presence or absence of the accommodation especially in younger patients in this series. Igarashi *et al.* stated that the manifest spherical equivalent was -0.44 ± 0.73 D at 8 years after ICL implantation^24^ Hersh *et al.* reported a mean regression of –1.14 ± 0.81 D in eyes requiring enhancement after myopic LASIK^25^ We also demonstrated that the manifest refraction of eyes requiring antiglaucoma drugs for myopic regression was –1.02 ± 0.52 D after myopic LASIK^26^ Therefore, in daily practice, both ICL implantation and LASIK for the correction of myopia tend to induce a myopic shift after surgery, and the mean amount of regression is relatively small (approximately up to -1D). Although we did not assess the clinical or optical effect of hyperopic defocus on visual acuity in these eyes, we believe that this information is useful both for the refractive surgeons concerned and for patients undergoing refractive surgery in a clinical setting. Our extensive search of the literature suggests that the present study is the first to investigate how myopic defocus affects visual acuity after the two surgical procedures concerned, from clinical and optical viewpoints. Sanders *et al.* previously demonstrated that the ICL had advantages over LASIK in eyes with moderate to high myopia as well as those with low myopia^14^,^15^ They also found ICL implantation to be superior to LASIK when the preoperative data matched, and when the degree of myopia lay between –3.00 to –7.88 D^16^ We previously reported that ICL implantation induced significantly fewer ocular HOAs than did wfg-LASIK for the correction of not only low to moderate myopia but also high myopia^17^,^18^ These findings suggest that ICL implantation may offer superior visual performance, compared with wfg-LASIK, in the correction of all degrees of myopia, although the superiority, in visual performance, of ICL implantation over wfg-LASIK for low to moderate myopia is not as great as than that for high myopia^17^,^18^ Buhren *et al.* reported a slight increase in the number of HOAs following iris-supported phakic IOL implantation^27^ It is suggested that both posterior chamber and iris-supported phakic IOL implantation cause fewer HOAs than wfg-LASIK, perhaps as a result of their preservation of the prolate shape of the cornea, irrespective of the degree of myopic correction. Moreover, there is less reduction of the retinal magnification of ICL implantation than in the case of wfg-LASIK^28^,^29^,^30^ Better visual performance after ICL implantation than after wfg-LASIK, even under myopic defocus conditions, may result from smaller increases in the number of HOAs and a smaller decrease in retinal magnification.

Contrary to our expectations, we found no significant differences in logMAR visual acuity between the two groups when there was a myopic defocus of -3 D, although the ICL group eyes had slightly better visual acuity than the wfg-LASIK group eyes. The negative effect of –3 D of myopic defocus on visual acuity is presumed to be much greater than the positive effect of either the smaller increase in HOAs or that in retinal magnification. This clinical finding was in agreement with our estimates of visual acuity by optical stimulation.

As for mechanisms causing myopic regression after LASIK, they are considered to include nuclear sclerosis of the crystalline lens, cornea1 ectasia, cornea1 hydration, stromal synthesis, compensatory epithelial hyperplasia, and axial length elongation^22^ ICL implantation requires only a 3-mm horizontal corneal incision, and so causes less alteration of the central cornea. Mechanisms for myopic regression after ICL implantation may therefore include nuclear sclerosis of the lens and axial length increase^31^

This study is burdened with at least two limitations: One is that we did not completely match the preoperative backgrounds of the patients, such as preoperative manifest refraction. The postoperative clinical outcomes were thought to be mainly influenced by preoperative refraction. However, the higher spherical equivalent refraction and the lower UDVA in the ICL group tended to bias the data in favor of the wfg-LASIK group, because it was often associated with poor safety and efficacy of the procedure. Therefore, we believe that this comparison is clinically reasonable for the assessment of the postoperative outcomes between the two surgical procedures. Another limitation is that we failed to use modern femtosecond laser technology for flap creation. Some studies have shown that the use of a femtosecond laser to perform LASIK leads to fewer HOAs than when a mechanical microkeratome is employed^32^,^33^,^34^,^35^,^36^ but other studies have demonstrated that no significant difference in HOAs normally arise between these two methods of flap creation^37^,^38^,^39^ Femtosecond laser flap creation may induce fewer HOAs than the mechanical microkeratome flap creation used in the current study. A further study employing femtosecond laser technology is necessary to confirm our preliminary findings.

In summary, our comparative study supports the view that visual performance after phakic IOL implantation was better than that after wfg-LASIK in a clinical setting, even in the presence of low to moderate myopic defocus. The results of optical simulation were seen to confirm these findings. It is suggested that phakic IOL implantation appears to offer superior visual performance, compared with wfg-LASIK, even when myopic regression occurs after surgery. We believe that the findings of the present investigation, despite their simplicity, help to provide a correct clinical understanding of the details of visual performance, especially, in ICL-implanted or post-wfg LASIK patients, when some degree of regression has taken place.

## Methods

Thirty eyes of 30 consecutive patients (13 men and 17 women) who underwent implantation of the posterior chamber phakic implantable collamer lens (Visian ICL^TM^, STAAR Surgical), and 30 eyes of 30 consecutive patients (13 men and 17 women) who underwent wfg-LASIK for the correction of myopia and myopic astigmatism, were included in this prospective study. The sample size in this study offered 94.0% statistical power at the 5% level in order to detect a 0.10-difference in logMAR visual acuity, when the standard deviation (SD) of the mean difference was 0.15. Keratoconic eyes were excluded from the study by using the keratoconus screening test of Placido disk videokeratography (TMS-2, Tomey, Nagoya, Japan). The study was approved by the Institutional Review Board of Kitasato University and followed the tenets of the Declaration of Helsinki. Informed consent was obtained from all patients after explanation of the nature and possible consequences of the study.

### Phakic Intraocular Lens Surgical Procedure

The patients preoperatively underwent 2 peripheral iridotomies with a neodymium-YAG laser. On the day of surgery, the patients were given dilating and cycloplegic agents. After topical anesthesia, a model V4b ICL (without a central hole) was inserted through a 3-mm clear corneal incision with the use of an injector cartridge (STAAR Surgical) after placement of a viscosurgical device (Opegan^TM^; Santen, Osaka, Japan) into the anterior chamber. The ICL was placed in the posterior chamber, the remaining viscosurgical device was completely washed out from the anterior chamber with balanced salt solution, and a miotic agent was instilled. For toric ICL implantation, to control for potential cyclotorsion in a supine position, the zero horizontal axis was marked preoperatively using a slit-lamp. After the ICL had then been placed in the posterior chamber and rotated by 22.5 degrees or less using the manipulator. Postoperatively, steroidal (0.1% betamethasone, Rinderon^TM^, Shionogi, Osaka, Japan) and antibiotic (0.3% levofloxacin, Cravit^TM^, Santen, Osaka, Japan) medications were administered topically 4 times daily for 2 weeks, and the dose was steadily reduced thereafter.

### Wavefront-guided Laser *in Situ* Keratomileusis Surgical Procedure

Wavefront-guided LASIK was performed with the Technolas 217z excimer laser system (Bausch & Lomb) to apply a flying spot of 1.0 or 2.0 mm in diameter with a Gaussian profile and a 120 Hz active eye tracker. The LSK-1 microkeratome (Moria, Antony, France) was utilized for creating a hinged corneal flap of 130-μm thickness. Postoperatively, steroidal (0.1% fluorometholone, Flumetholone ^TM^, Santen) and antibiotic (0.3% levofloxacin, Cravit^TM^, Santen) medications were topically administered 4 times daily for 2 weeks.

### Assessment of Visual Acuity under Myopic Defocus

After we fully corrected manifest refraction, we randomly assessed logMAR visual acuity under myopic defocus of 0 to -3 D (1D step) in the ICL and wfg-LASIK groups, under the two monocular (non-cycloplegic and cycloplegic) conditions. Visual acuity measurement was performed using a Snellen chart with Japanese letters at a distance of 5 m with best correction (not with habitual correction). Refraction was measured by an optometrist using an automated refractometer (ARK-700A, Nidek, Gamagori, Japan), and the results were used as a starting point for a full manifest refraction.

In the subgroup analysis, thereafter, cycloplegia was achieved with 3 drops of 1% cyclopentolate hydrochloride (Cyplegin^TM^, Santen) in 20 eyes of 20 patients in each group, spaced 5 minutes apart. Autorefraction (ARK-700A) was undertaken at least 30 minutes after the third administration of cyclopentolate hydrochloride and only if the pupillary light reflex was absent. After we fully corrected cycloplegic refraction, we also randomly assessed logMAR visual acuity under myopic defocus of 0 to -3 D (1D step) using a 3-mm artificial pupils in these eyes. All examinations were performed by experienced ophthalmic technicians who masked to the treatment.

### Optical Simulation

For the estimation of visual acuity by optical simulation, ray tracing was carried out using ZEMAX optical design software (ZEMAX Corp., Bellevue, WA, USA). A modified Liou-Brennan model eye was used for the simulation^40^ The model eye was designed with realistic factors such as a centered optical system, corneal asphericity, an iris pupil, a Stiles-Crawford effect, an IOL, and chromatic aberration. A myopic model of -10 D was made by optimizing the vitreous length. The basic anterior and posterior curvature, thickness, axial position, and refractive index of the ICL were 7.70 mm, −7.70 mm, 1.03 mm, 5.03 mm, and 1.413, respectively. The same model, except for the anterior cornea, was used for the simulation of wfg-LASIK. The ICL power used for the simulation was -11.90 D in aqueous humor (equivalent to -10 D at the corneal plane). The amount of refractive correction in the anterior cornea of wfg-LASIK was 10.0 D. The preoperative and postoperative wfg-LASIK corneal aberration data were used for the ICL implantation and wfg-LASIK simulations, respectively. Myopic defocus in the eye model ranged from 0 to -3 D in 1 D step, which were adjusted by changing the corneal curvature by using biconvex spherical eye glass model. The size of the entrance pupil for the simulation was 3.0 mm. Calculation of the modulation transfer function (MTF), which is the ratio of the image contrast to object contrast as a function of spatial frequency, was performed by using the ZEMAX. Visual acuities ranging from 0 to -3 D in 1 D step were estimated from the MTF curve and Campbell & Green’s retinal threshold curve^41^ The visual target for which the modulation is below the threshold of the spatial frequency corresponding to that visual target will not be resolved. Therefore, the intersection between MTF and the threshold is the estimated visual acuity^42^ The estimated logMAR visual acuities in sagittal and tangential directions were averaged. Additionally, our results contained the effect of spurious resolution^43^ Spurious resolution is a phenomenon in which MTF falls to zero as the defocus level is increased.

### Statistical Analysis

All statistical analyses were performed using a commercially available statistical software (Ekuseru-Toukei 2010, Social Survey Research Information Co, Ltd., Tokyo, Japan). One-way analysis of variance (ANOVA) was used for the analysis of the time course of changes, the Dunnett test being employed for multiple comparisons. The Mann-Whitney U test was used to compare the data between the two groups. The results are expressed as mean ± SD, and a value of p < 0.05 was considered statistically significant.

## Author Contributions

K.K., K.S., A.I. and T.K. designed the study, K.K., A.I. and T.K. acquired and analysed the data. K.K., K.S., A.I. and T.K. discussed the results. K.K. drafted the manuscript. K.S., A.I. and T.K. critically revised the manuscript.

## Additional Information

**How to cite this article**: Kamiya, K. *et al.* Effect of Myopic Defocus on Visual Acuity after Phakic Intraocular Lens Implantation and Wavefront-guided Laser *in Situ* Keratomileusis. *Sci. Rep.* **5**, 10456; doi: 10.1038/srep10456 (2015).

## Figures and Tables

**Table 1:** Patient demographics in eyes undergoing implantable collamer lens implantation and wavefront-guided laser *in situ* keratomileusis.

	**ICL group**	**Wavefront-guided LASIK group**	***P*** **value**
Age	30.1 ± 5.8 (range, 23 to 39 years)	31.4 ± 5.2 (range, 19 to 39 years)	0.27
Gender (% female)	62.5	76.4	0.38
Preoperative logMAR UDVA	1.38 ± 0.20 (range, 1.00 to 1.70)	1.11 ± 0.21 (range, 0.52 to 1.52)	< 0.001
Preoperative logMAR CDVA	–0.21 ± 0.06 (range, –0.30 to –0.18)	–0.19 ± 0.09 (range, –0.30 to 0.00)	0.53
Preoperative manifest spherical equivalent (D)	–7.92 ± 1.94 (range, -4.00 to –11.75)	–4.90 ± 1.95 (range, –2.00 to –9.75)	<0.001
Preoperative manifest cylinder (D)	–1.03 ± 0.83 (range, –3.00 to 0.00)	–0.84 ± 0.79 (range, –3.00 to 0.00)	0.41
Postoperative logMAR UDVA	–0.19 ± 0.11 (range, –0.30 to 0.05)	–0.14 ± 0.11 (range, –0.30 to 0.10)	0.11
Postoperative logMAR CDVA	–0.25 ± 0.08 (range, –0.30 to –0.08)	–0.20 ± 0.07 (range, –0.30 to –0.08)	0.01
Preoperative manifest spherical equivalent (D)	–0.05 ± 0.18 (range, –0.50 to 0.25)	–0.02 ± 0.37 (range, –1.00 to 0.88)	0.98
Preoperative manifest cylinder (D)	–0.38 ± 0.38 (range, –1.25 to 0.00)	–0.35 ± 0.31 (range, –1.00 to 0.00)	0.94

ICL=implantable collamer lens; LASIK=laser *in situ* keratomileusis; D=diopters; logMAR=logarithm of the minimal angle of resolution; UDVA=uncorrected distance visual acuity; CDVA=corrected distance visual acuity.
